# A novel conceptual model of heart rate autonomic modulation based on a small-world modular structure of the sinoatrial node

**DOI:** 10.3389/fphys.2023.1276023

**Published:** 2023-12-11

**Authors:** Alexander V. Maltsev, Michael D. Stern, Edward G. Lakatta, Victor A. Maltsev

**Affiliations:** Intramural Research Program, National Institute on Aging, Baltimore, MD, United States

**Keywords:** heartbeat, sinoatrial node, pacemaker mechanism, autonomic modulation of heart rate, shift of the leading pacemaker site, small-world network, cell heterogeneity, numerical model

## Abstract

The present view on heartbeat initiation is that a primary pacemaker cell or a group of cells in the sinoatrial node (SAN) center paces the rest of the SAN and the atria. However, recent high-resolution imaging studies show a more complex paradigm of SAN function that emerges from heterogeneous signaling, mimicking brain cytoarchitecture and function. Here, we developed and tested a new conceptual numerical model of SAN organized similarly to brain networks featuring a modular structure with small-world topology. In our model, a lower rate module leads action potential (AP) firing in the basal state and during parasympathetic stimulation, whereas a higher rate module leads during β-adrenergic stimulation. Such a system reproduces the respective shift of the leading pacemaker site observed experimentally and a wide range of rate modulation and robust function while conserving energy. Since experimental studies found functional modules at different scales, from a few cells up to the highest scale of the superior and inferior SAN, the SAN appears to feature hierarchical modularity, i.e., within each module, there is a set of sub-modules, like in the brain, exhibiting greater robustness, adaptivity, and evolvability of network function. In this perspective, our model offers a new mainframe for interpreting new data on heterogeneous signaling in the SAN at different scales, providing new insights into cardiac pacemaker function and SAN-related cardiac arrhythmias in aging and disease.

## Introduction

The sinoatrial node (SAN) is the primary pacemaker of the heart, generating rhythmic electrical impulses that drive heart contractions at rates that satisfy blood supply under given conditions. Since the successful application of Hodgkin–Huxley theory to cardiac cells (Noble, 1960), the cardiac pacemaker field had long been dominated by the idea that the ensemble of membrane ion currents [dubbed later as the membrane clock (Maltsev and Lakatta, 2009)] in pacemaker cells drives their spontaneous diastolic depolarization and spontaneous action potential (AP) firing. Primary pacemaker cells in the SAN center were thought to dictate the excitation rate of other SAN cells (Sano et al., 1978; Bleeker et al., 1980). Thus, extensive search for the mechanisms of autonomic modulation of the heart rate had initially been mainly limited to respective modulation of specific membrane ion currents, such as *I*_CaL_, *I*_K_, *I*_f_, and *I*_KACh,_ in single-cell models (Hauswirth et al., 1969; Brown et al., 1975; Noma and Trautwein, 1978; Brown et al., 1979; DiFrancesco et al., 1989; Demir et al., 1999; Zhang et al., 2002; Himeno et al., 2008; Tao et al., 2011). The critical importance of oscillatory local Ca releases (Ca clock) for the β-adrenergic receptor (βAR) and cholinergic receptor (ChR) stimulations has been demonstrated by live-cell confocal microscopy imaging (Vinogradova et al., 2002; Lyashkov et al., 2009). Thus, the search for numerical formulations for autonomic modulation mechanisms included the coupled-clock function, but still within the single-cell paradigm (Vinogradova et al., 2006; Lyashkov et al., 2009; Maltsev and Lakatta, 2010; 2013).

The SAN cell population, however, is extremely heterogeneous with respect to cell shape, size, and biophysical properties. The expression of Ca-cycling proteins and membrane ion channels varies substantially among individual SAN cells (Honjo et al., 1996; Honjo et al., 1999; Lei et al., 2001; Musa et al., 2002; Monfredi et al., 2018). For example, *I*_*CaL*_ density varies by an order of magnitude (Monfredi et al., 2018) and cells differ dramatically in their response to autonomic modulation (Kim et al., 2021; Yang et al., 2021). Furthermore, some cells isolated from the SAN (dubbed dormant cells) do not generate spontaneous AP firing but can generate rhythmic APs during βAR stimulation (Kim et al., 2018; Louradour et al., 2022). Cells isolated from the superior or inferior SAN also exhibit different automaticity (Grainger et al., 2021). Cell clusters isolated from different SAN regions also showed different AP characteristics and responses to the βAR and ChR stimulations (Kodama and Boyett, 1985; Opthof et al., 1987).

Heterogeneous cell properties are in agreement with the recent results of single-cell-resolution imaging of intact SAN tissues. These studies demonstrated that while the majority of SAN cells indeed fire synchronously with a common period, many cells fire at various rates and irregularly, or remain silent, generating only local Ca releases (Bychkov et al., 2020; Fenske et al., 2020), like dormant cells discovered previously in single-cell studies. The number of silent cells in tissues was increased by ChR stimulation (Fenske et al., 2020). The tissue excitation within the SAN center appeared to be discontinuous, consisting of functional cell clusters with different firing patterns (Bychkov et al., 2020). Functional modules were later revealed in these SAN data by random matrix theory and PCA (Norris and Maltsev, 2023). Application of the neurobiochemical marker S100B (Ca-binding protein-B) to the SAN desynchronized Ca signaling and thereby revealed individual cell clusters operating at lower rates (Bychkov et al., 2022). Overall, synchronized AP firing seems to emerge from heterogeneous signaling with the heart’s pacemaker mimicking brain cytoarchitecture and function, thereby resembling the multiscale complex processes of impulse generation within the clusters of neurons in neuronal networks (Bychkov et al., 2020; Bychkov et al., 2022).

On a larger scale, the entire SAN exhibits two spatially distinct competing pacemaker regions, superior and inferior SANs (termed sSAN and iSAN, respectively) (Brennan et al., 2020). Those regions feature different profiles of expression of ion channels, cardiac receptors, neural proteins, and transcription factors and preferentially control the fast and slow heart rates via autonomic nervous system modulation that is accompanied by the respective shift of leading pacemaker locations (Brennan et al., 2020). There is also evidence of activation of different exit pathways (in the tail, center, and head of the SAN) under different conditions, which could correspond to more cell clusters or pacemaker regions (Li et al., 2017; Li et al., 2020).

Thus, while single-cell models are capable of reproducing the entire range of the heart rate of most mammals and a tissue composed of them would do the same, additional pacemaker mechanisms seem to emerge at the tissue level. These complex mechanisms merit future studies, both theoretical and experimental. A large variety of SAN tissue models have been developed, and these tissue models were able to reproduce the pacemaker shift caused by autonomic modulation, e.g., due to different sensitivities to ACh (Michaels et al., 1987). The SAN tissue in those models was populated by heterogeneous cells either randomly (Michaels et al., 1987; Syunyaev and Aliev, 2011; Campana et al., 2022; Moise and Weinberg, 2023) or following a gradient distribution (Huang et al., 2011; Muñoz et al., 2011; Inada et al., 2014). Based on the existing experimental evidence, the importance of gradients in SAN cellular properties cannot be ruled out. On the other hand, the shift is likely “linked to the presence of distinct anatomically and functionally defined intranodal pacemaker clusters that are responsible for the generation of the heart rhythm at different rates,” as recently hypothesized by Lang and Glukhov (2021) based on substantial experimental data in mouse, rabbit, canine, and human SANs.

Existing SAN tissue models do not include pacemaker cell clusters and complex brain-like signaling observed experimentally. While small clusters of cells can emerge spontaneously in randomly distributed cells (Maltsev et al., 2022a; Campana et al., 2022; Moise and Weinberg, 2023), likely due to Poisson clumping (Aldous, 1989), the specialized pacemaker clusters can actually be much larger, e.g., sSAN and iSAN (Brennan et al., 2020), and, as such, emerge via morphogenesis rather than randomly.

Here, we propose a novel conceptual numerical model of heart rate autonomic modulation based on recent experimental studies on SAN cells in tissues. Our model simulates the heterogeneous SAN as a network of loosely connected clusters (modules or hubs) of tightly coupled cells, featuring clustered small-world topology, like in brain networks (Russo et al., 2014; Bassett and Bullmore, 2017). A small-world network is a type of network in which most nodes are not neighbors of one another, but most nodes can be reached from every other node by a small number of steps. In our simple model consisting of two modules, each module specializes in driving its specific rate range: a higher rate module (HRM) leads AP firing in the system during βAR stimulation, while a lower rate module (LRM) leads in the basal state and during ChR stimulation.

## Materials and methods

### Single-cell model

Each pacemaker cell in our multicellular SAN model is simulated by the Maltsev–Lakatta model of the central SAN cells of rabbits, having a coupled-clock pacemaker mechanism (Maltsev and Lakatta, 2009). The CellML computer code for the model (at http://models.cellml.org/workspace/maltsev_2009) can be executed via Cellular Open Resource (COR, http://www.opencor.ws/). The Supplementary text provides further details, including our formulations of βAR and ChR stimulation. Supplementary Table S1 provides the initial conditions.

### Developing a new multicellular model

**FIGURE 1:**
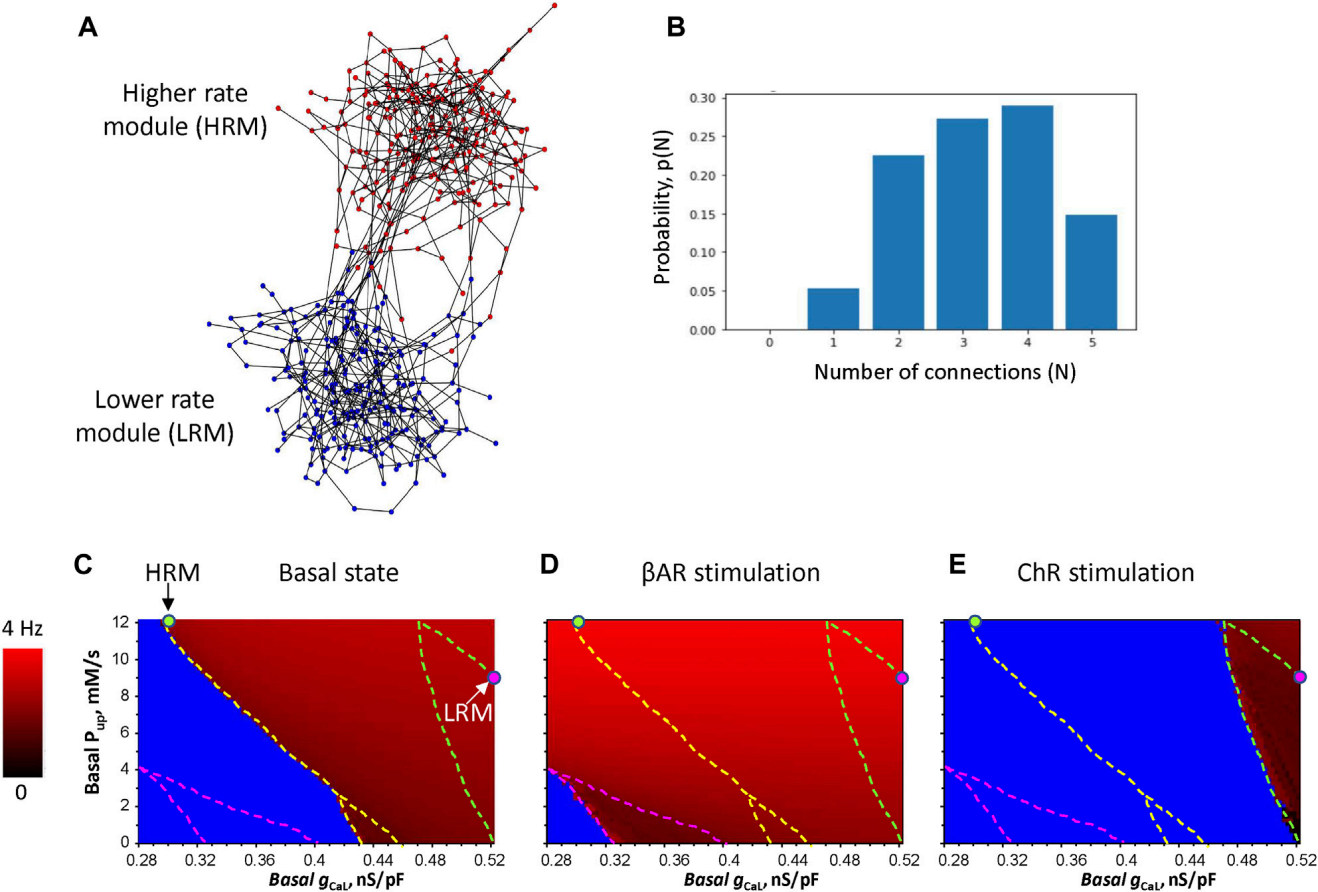
Development of the 3D model of SAN tissues with brain-like modular structure and function. **(A)** An example of the model, which consists of two modules. Nodes in the network designate cells, and the edges imply electrical coupling between the cells: 242 HRM cells (red circles) and 243 LRM cells (blue circles), each connection has the intracellular resistivity ρ = 3,750 MΩ*m. The interactive 3D view of the model is provided in Supplementary Figure S2. **(B)** Probability distribution of the number of connections between cells in the SAN model in panel **(A)**. **(C–E)** Results of the parametric sensitivity analysis of βAR and ChR stimulation in individual isolated SAN cells with respect to the basal conductance of *I*_CaL_ (*g*_CaL_, *x*-axis) and sarcoplasmic reticulum Ca pumping rate (*P*_up_, *y*-axis). The image shows the result of 97 (*x*)*61 (*y*) = 5,917 simulations (each pixel = 0.0025 nS/pF * 0.2 mM/s). The fork-like dash lines outline the bifurcation borders between non-firing (blue), firing (gradual red shades), and chaotic firing (mosaic red shades within the lower part of the fork). Red shades code AP firing rates from 0 (black) to 4 Hz (pure red). The HRM and LRM were populated with cells with parameters marked by green and magenta circles, respectively.

Our new iterative algorithm generates a modular SAN network based on a saw-shaped probability density function of edges, with the peaks designating attractor points (see Supplementary Figure S1 for details and our Python code at https://github.com/alexmaltsev/IterativeNetworkGenerator). Here, we investigated an example of such a SAN system consisting of two modules (Figure 1A; Supplementary Figure S2 in the interactive 3D viewer). While the exact connectome for the SAN remains unknown, our algorithm was tuned to generate cellular networks with a reasonable number of connections between neighboring cells ranging from 1 to 5 (Figure 1B) based on the visual inspection of previously reported high-resolution images of SAN tissues (Bychkov et al., 2020; Bychkov et al., 2022). We also calculated the small-world coefficient that compares the clustering and path length of a given network to an equivalent random network with the same node degree (number of connections) on average: *σ = (C/C*_*r*_ *)/(L/L*_*r*_ *)* (Humphries and Gurney, 2008). When *σ > 1*, the network is effectively small-world. Our network example (Figure 1A) had *σ = 3*.*62* calculated using Python libraries *average_clustering* and *average_shortest_path_length*. Model simulations were performed using NVIDIA RTX A6000 GPU as previously described (Maltsev et al., 2022a), based on the original CUDA-C algorithm proposed by Campana (2015).

## Results

### Parametric sensitivity analyses

The cell parameters were differentially set among the two modules (HRM and LRM), but identical within each module, tuned to generate specific rates. The LRM had ion currents and Ca cycling to generate lower physiological AP rates in the basal state and during ChR stimulation. The HRM had parameters to generate physiological AP firing rates during βAR stimulation. We performed wide-range sensitivity analyses of isolated cell function (Figures 1C–E) with respect to two key model parameters, basal *g*_CaL_ determining maximal *I*_CaL_ conductance and basal *P*_up_ determining maximal sarcoplasmic reticulum Ca pumping rate. The analysis of the basal state, reported first by Maltsev and Lakatta (2009), was complemented by two new analyses for βAR and ChR stimulations.

### Autonomic modulation of the system

The idea to create efficient rate modulation by the system is based on the interplay of membrane clock and Ca clocks in different modules. The LRM should generate a higher basal rate (and safe function during ChR stimulation) due to a substantially stronger membrane clock, but during βAR stimulation, its rate increase should be smaller due to its weaker Ca clock. In contrast, the HRM should have a stronger Ca clock so that the effect of βAR stimulation will be stronger, but the basal rate will be lower due to a weaker membrane clock (i.e., smaller *g*_*CaL*_). We were able to find such system parameters in the *P*_up_ *-g*_CaL_ diagrams. LRM cells have a larger *g*_CaL_ = 0.52 nS/pF but smaller *P*_up_ = 9 mM/s (magenta circle) in close proximity to the bifurcation border to generate the lowest possible AP rate without arrhythmia during ChR stimulation. HRM cells, in turn, have a smaller *g*_CaL_ = 0.3 nS/pF but larger *P*_up_ = 12 mM/s (green circle) also in close proximity to the bifurcation line, allowing the LRM to lead in the basal state.

**FIGURE 2:**
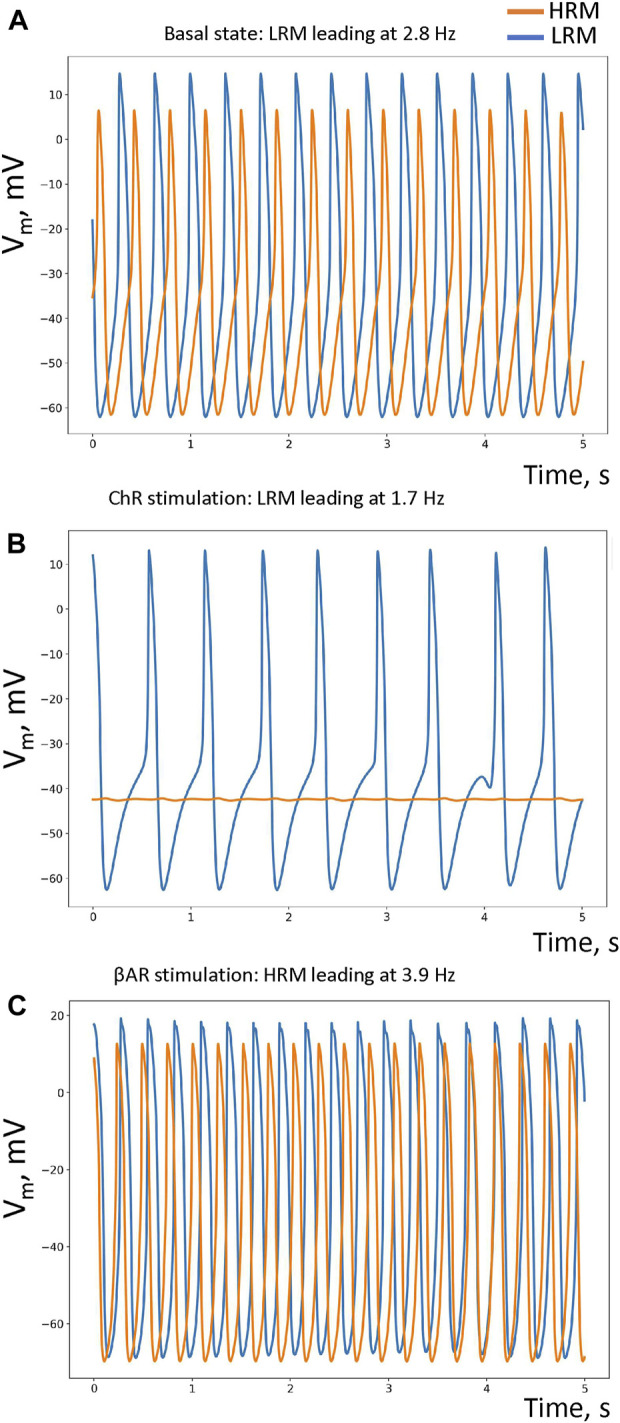
Performance of our small-world SAN model under different conditions. **(A–C)** Simulated AP traces in, respectively, the basal state (in the absence of autonomic modulation), in ChR stimulation, and βAR stimulation in each pacemaker module: HRM (orange traces) and LRM (blue traces). The respective leading/pacing rates are shown at the top of each panel. Histograms of the average AP cycle length of each cell in each module under each condition are provided in Supplementary Figure S3.

The LRM led in the baseline with an AP cycle length of 359.74 ± 0.18 ms (mean ± SD, among cells), while the HRM fired APs with a delay and a longer mean cycle length of 365.48 ± 53.46 ms (Figure 2A; Supplementary Video S1, respective histograms in Supplementary Figure S3). During ChR stimulation, the LRM remained leading (cycle length = 585.19 ± 22.73 ms), but the HRM ceased firing (Figure 2B; Supplementary Video S2). During βAR stimulation, the leading module was the HRM (mean cycle length = 256.69 ± 0.09 ms), while the LRM fired APs with a substantial delay and longer cycle length (272.95 ± 3.25 ms) (Figure 2C; Supplementary Video S3). Thus, the modular system generated AP rates within a wide range from 1.7 to 3.9 Hz by two loosely coupled modules, each of which was tuned for different rates.

It is important to note that our method of building the SAN network involves a stochastic step (Supplementary Figure S1). Thus, to further support our model results, we generated two more model realizations (Supplementary Figure S4). Our simulation results using these additional realizations were similar to those described above.

## Discussion

The existing numerical models of autonomic modulation of the heart rate do not capture the modern view on the SAN structure–function relationship gleaned from experimental studies, i.e., distinct anatomically and functionally defined intranodal pacemaker clusters generate the heartbeats at different rates (Brennan et al., 2020; Bychkov et al., 2020; Lang and Glukhov, 2021). Here, for the first time, we provide a numerical validation of the concept that the entire range of autonomic modulation of the heart rate can be achieved via a brain-like structure featuring loosely connected clusters (functional modules) of tightly coupled cells, specialized for particular AP firing rates.

Based on these considerations and our new model simulations, the role of parasympathetic stimulation in the new emerging paradigm of heart rate modulation is not just about decreasing the AP rate of the pacemaker cell *per se* but also about suppressing the activity of module(s) generating higher AP rates in order to unmask the function of other module(s) that are tuned to safely operate at lower rates. Thus, the SAN pacemaker system is modulated via distinct cell clusters (Brennan et al., 2020; Bychkov et al., 2020; Lang and Glukhov, 2021), similar to switching car gears to achieve the best performance at different speeds under different conditions. In these terms, ChR stimulation acts as a powerful downshifter, overriding βAR stimulation in agreement with well-known phenomena known as the anti-adrenergic effect (Belardinelli et al., 1995; Norton et al., 1999), i.e., stronger ACh and adenosine effects in the presence of βAR stimulation, as well as accentuated antagonism, i.e., substantially smaller sympathetic heart rate effects at high levels of vagal tone (Levy, 1984; Mizuno et al., 2008).

For the most effective modulation (broadest rate range), our system operates at the edge of criticality, i.e., close to the bifurcation line (Figures 1C–E). Many biological systems benefit from operating at the edge of criticality to reap benefits such as “an optimal balance between robustness against perturbations and flexibility to adapt to changing conditions” (Muñoz, 2018). Another benefit of the modular SAN system is energy saving. Indeed, while the LRM generates rare APs during ChR stimulation, the HRM ceased firing (becoming “dormant”), reproducing experimental results that a notable SAN cell population becomes dormant in the presence of parasympathetic stimulation (Fenske et al., 2020). These dormant cells consume much less energy in the absence of AP firing.

With respect to limitations and future studies, we present here a simple conceptual model of the modular SAN, assuming that numerous details of anatomical structures and heterogeneity of cell biophysical properties (Honjo et al., 1996; Honjo et al., 1999; Lei et al., 2001; Musa et al., 2002; Monfredi et al., 2018) within modules will be implemented in the future. We note that different contributions of Ca and membrane clocks in the modules (Figures 1C–E) are just one logical possibility to construct a modular SAN system with effective, realistic operation. However, the exact distributions of *g*_*CaL*_ and *P*_*up*_ in SAN tissues remain unknown, which merits further investigation along with many other important parameters of cells and their connections. A major model development could be the implementation of heterogeneous, local Ca releases (Stern et al., 2014; Maltsev et al., 2022b) as well as different profiles of local expression of ion channels, cardiac receptors, neural proteins, and transcription factors in different regions of the SAN (Brennan et al., 2020).

The next important step will be to quantify the SAN connectome and physiome to generate more realistic 3D structures of the functional modules. In a broader context, “brain networks and many other complex systems demonstrate the property of hierarchical modularity, or modularity on several topological scales: within each module, there will be a set of sub-modules and within each sub-module, there will be a set of sub-sub-modules, etc. There are several general advantages to modular and hierarchically modular network organization, including greater robustness, adaptivity, and evolvability of network function” (Meunier et al., 2010). Following the idea of hierarchical modularity, sSAN and iSAN (Brennan et al., 2020) would represent the highest hierarchy of SAN tissues, whereas they, themselves, may contain sub-modules, like in the brain. Indeed, module sizes span from a few cells (Bychkov et al., 2020; Norris and Maltsev, 2023) up to 5,000 cells (Bleeker et al., 1980), and the idea of the hierarchical modularity of the SAN can be explored in future studies. Future models consisting of more than two modules (Supplementary Figure S5) will be able to explain the shift to multiple locations within the entire SAN in response to various stimuli (including changes in pH, temperature, K^+^, and ion channel blockers). Small-world structures have special features that allow for the performance of complex functions, like in the brain, including long-term correlations and memory, lacking in simple structures (e.g., gradient and mosaic SAN models). Thus, future SAN models with complex modular structures will also provide new insights into the mechanisms of heart rate variability and its complexity.

It will also be important to test model behavior when the number of connections and their resistivities is varied. Further model development will add cellular links to the CX43+ network and the SAN exits. An interesting hypothesis could be then that each such exit is associated with the respective module that is fine-tuned for a particular rate range. Furthermore, the system of loosely coupled clusters is likely less disposed to reentrant arrhythmias due to its very structure featuring fewer reentrant pathways among the modules. A new mechanism of SAN operation is via a percolation phase transition (Weiss and Qu, 2020). In our model, such percolation would easily occur within each cluster of tightly connected cells but not through all loosely connected clusters, keeping their fine-tuned specific rates intact, thereby supporting the robust and flexible operation of the system.

The SAN is controlled by the so-called “heart’s little brain” (Armour, 2008; Herring and Paterson, 2021), and the entire SAN system includes many cell types. Recent studies identified autonomic plexus, peripheral glial cell web, and a novel S100B(+)/GFAP (–) interstitial cell type embedded within the HCN4+ cell meshwork that increase the structural and functional complexity of the SAN and provide a new regulatory pathway of rhythmogenesis (Bychkov et al., 2022). Thus, additional layers of cellular networks of different natures can be added to our new model and tested with respect to their roles in tuning and maintaining the structure and function of the modular system at its optimal performance.

In summary, the modular SAN structure discovered by recent experimental studies represents the frontier of pacemaker research. The new experimental data on SAN heterogeneity can be included in the new framework proposed in this study, and novel mechanistic insights can be revealed by further testing *in silico* the roles and complex interplay of particular components within the modular system. System behavior can be analyzed via wide-range multi-component sensitivity analyses, bifurcation analysis, information flow analysis (used in neuroscience), etc. This will test numerous hypotheses and interpret new data on SAN heterogeneities in funny current, K currents, Ca current (e.g., via Cav1.2 and Cav1.3), Na/Ca exchanger, ryanodine and IP3 receptors, Ca-release activated channels, etc. The new framework will also be helpful for interpreting new data on SAN heterogeneities in cell connections (e.g., connexins), SAN cell network structure (i.e., connectome, like in neuronal networks), and interactions with networks of other cell types. Future model development will also include connections of the modules to SAN periphery and the atria, providing new insights on robust impulse conduction.

## Data Availability

The raw data supporting the conclusion of this article will be made available by the authors, without undue reservation.
