# Supplementary material for: A novel conceptual model of heart rate autonomic modulation based on a small-world modular structure of the sinoatrial node
Source: Front Physiol. 2023 Dec 11;14:1276023. doi: 10.3389/fphys.2023.1276023 (PMC10750401; doi:10.3389/fphys.2023.1276023)
Supplement: Supplementary file 2 [file Presentation1.PPTX]

## Slide 1
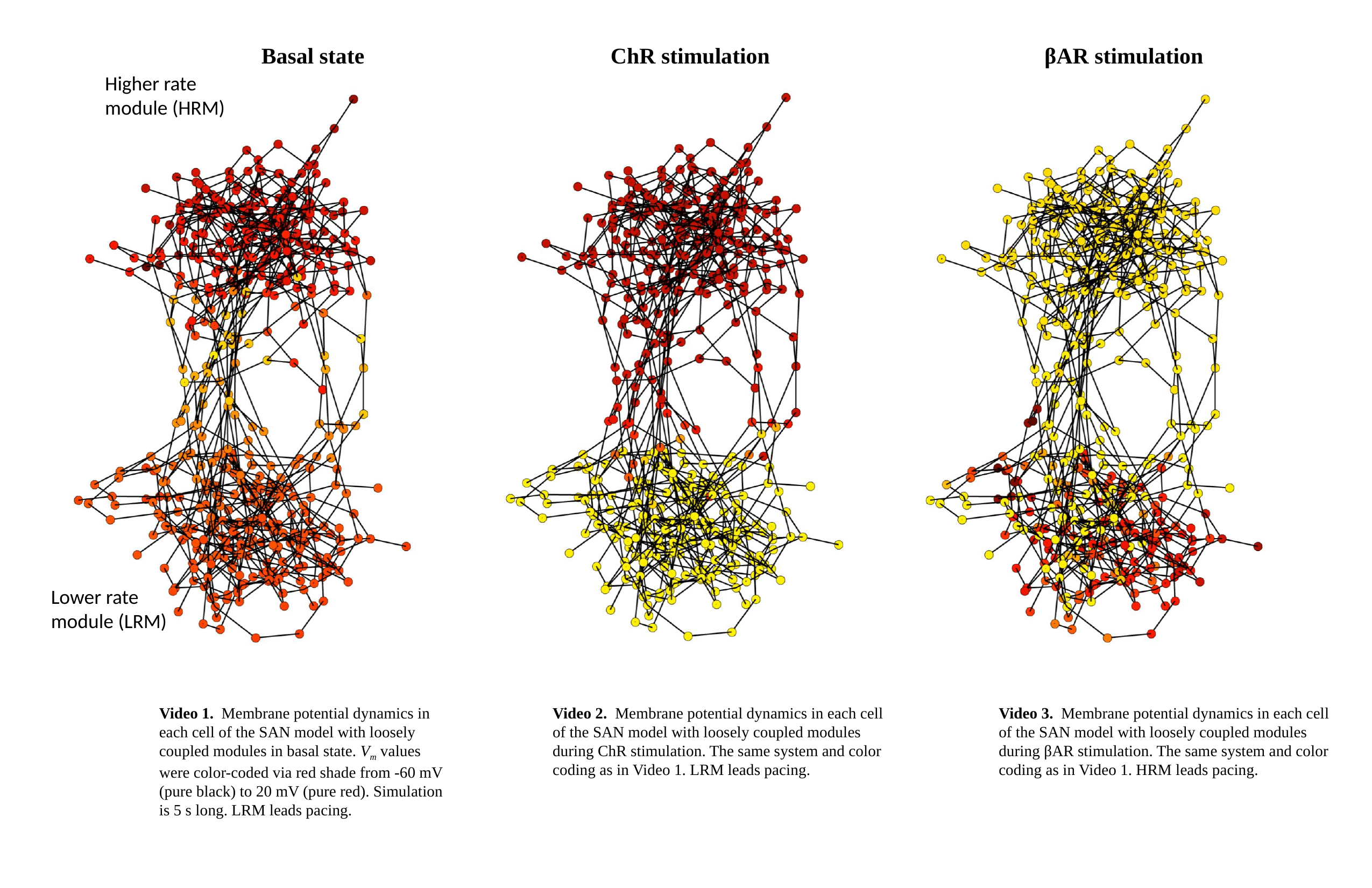

Basal state
ChR stimulation
βAR stimulation
Higher rate module (HRM)
Lower rate module (LRM)
Video 1. Membrane potential dynamics in each cell of the SAN model with loosely coupled modules in basal state. Vm values were color-coded via red shade from -60 mV (pure black) to 20 mV (pure red). Simulation is 5 s long. LRM leads pacing.
Video 2. Membrane potential dynamics in each cell of the SAN model with loosely coupled modules during ChR stimulation. The same system and color coding as in Video 1. LRM leads pacing.
Video 3. Membrane potential dynamics in each cell of the SAN model with loosely coupled modules during βAR stimulation. The same system and color coding as in Video 1. HRM leads pacing.
