# Supplementary material for: A novel conceptual model of heart rate autonomic modulation based on a small-world modular structure of the sinoatrial node
Source: Front Physiol. 2023 Dec 11;14:1276023. doi: 10.3389/fphys.2023.1276023 (PMC10750401; doi:10.3389/fphys.2023.1276023)
Supplement: Supplementary file 3 [file Presentation2.PPTX]

## Slide 1
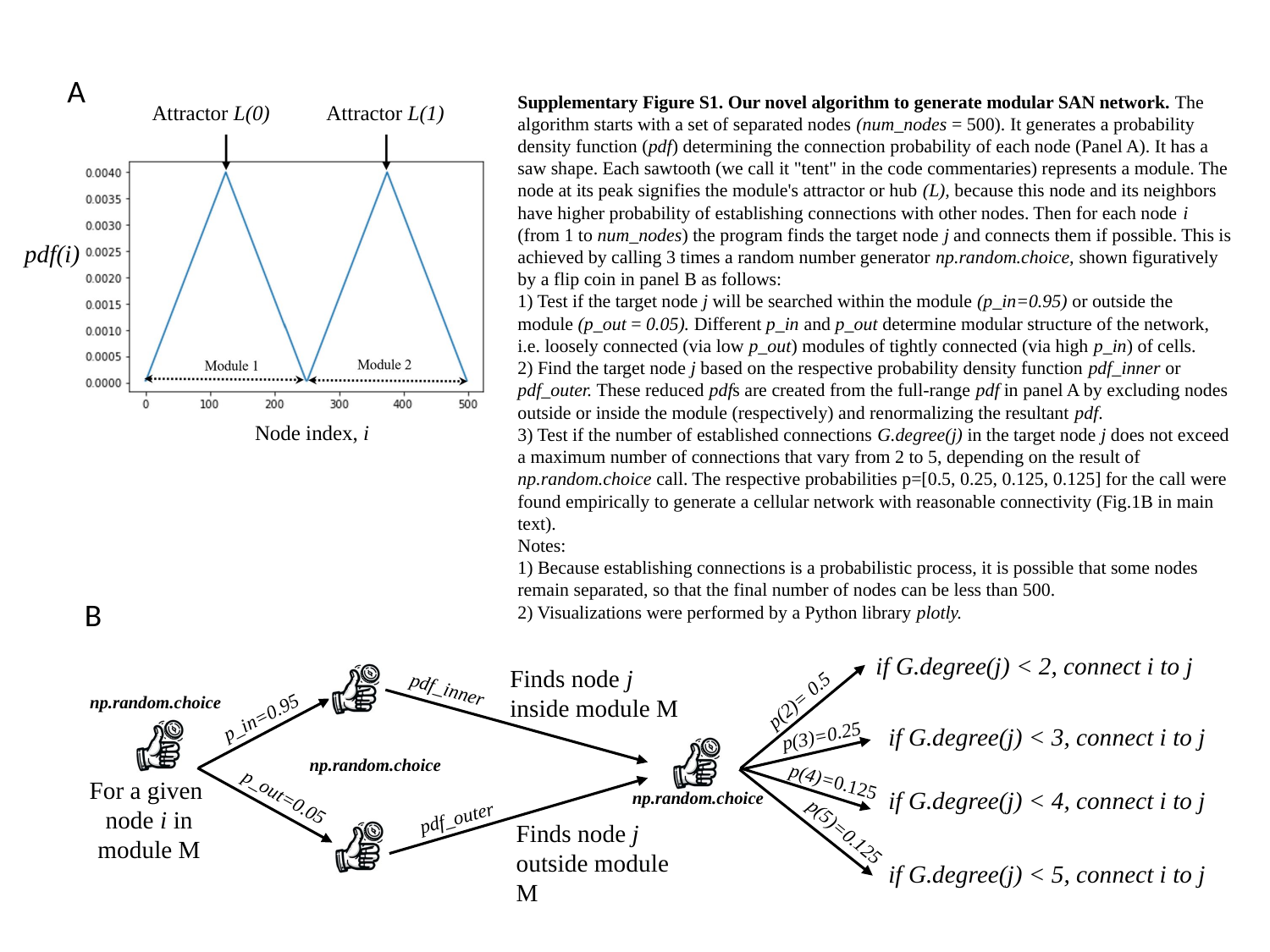

A
Supplementary Figure S1. Our novel algorithm to generate modular SAN network. The algorithm starts with a set of separated nodes (num_nodes = 500). It generates a probability density function (pdf) determining the connection probability of each node (Panel A). It has a saw shape. Each sawtooth (we call it "tent" in the code commentaries) represents a module. The node at its peak signifies the module's attractor or hub (L), because this node and its neighbors have higher probability of establishing connections with other nodes. Then for each node i (from 1 to num_nodes) the program finds the target node j and connects them if possible. This is achieved by calling 3 times a random number generator np.random.choice, shown figuratively by a flip coin in panel B as follows:
1) Test if the target node j will be searched within the module (p_in=0.95) or outside the module (p_out = 0.05). Different p_in and p_out determine modular structure of the network, i.e. loosely connected (via low p_out) modules of tightly connected (via high p_in) of cells.
2) Find the target node j based on the respective probability density function pdf_inner or pdf_outer. These reduced pdfs are created from the full-range pdf in panel A by excluding nodes outside or inside the module (respectively) and renormalizing the resultant pdf.
3) Test if the number of established connections G.degree(j) in the target node j does not exceed a maximum number of connections that vary from 2 to 5, depending on the result of np.random.choice call. The respective probabilities p=[0.5, 0.25, 0.125, 0.125] for the call were found empirically to generate a cellular network with reasonable connectivity (Fig.1B in main text).
Notes:
1) Because establishing connections is a probabilistic process, it is possible that some nodes remain separated, so that the final number of nodes can be less than 500.
2) Visualizations were performed by a Python library plotly.
Attractor L(0)
Attractor L(1)
pdf(i)
Node index, i
B
if G.degree(j) < 2, connect i to j
Finds node j inside module M
pdf_inner
p(2)= 0.5
np.random.choice
p_in=0.95
if G.degree(j) < 3, connect i to j
p(3)=0.25
np.random.choice
p(4)=0.125
For a given
node i in module M
if G.degree(j) < 4, connect i to j
p_out=0.05
np.random.choice
pdf_outer
Finds node j outside module M
p(5)=0.125
if G.degree(j) < 5, connect i to j

## Slide 2
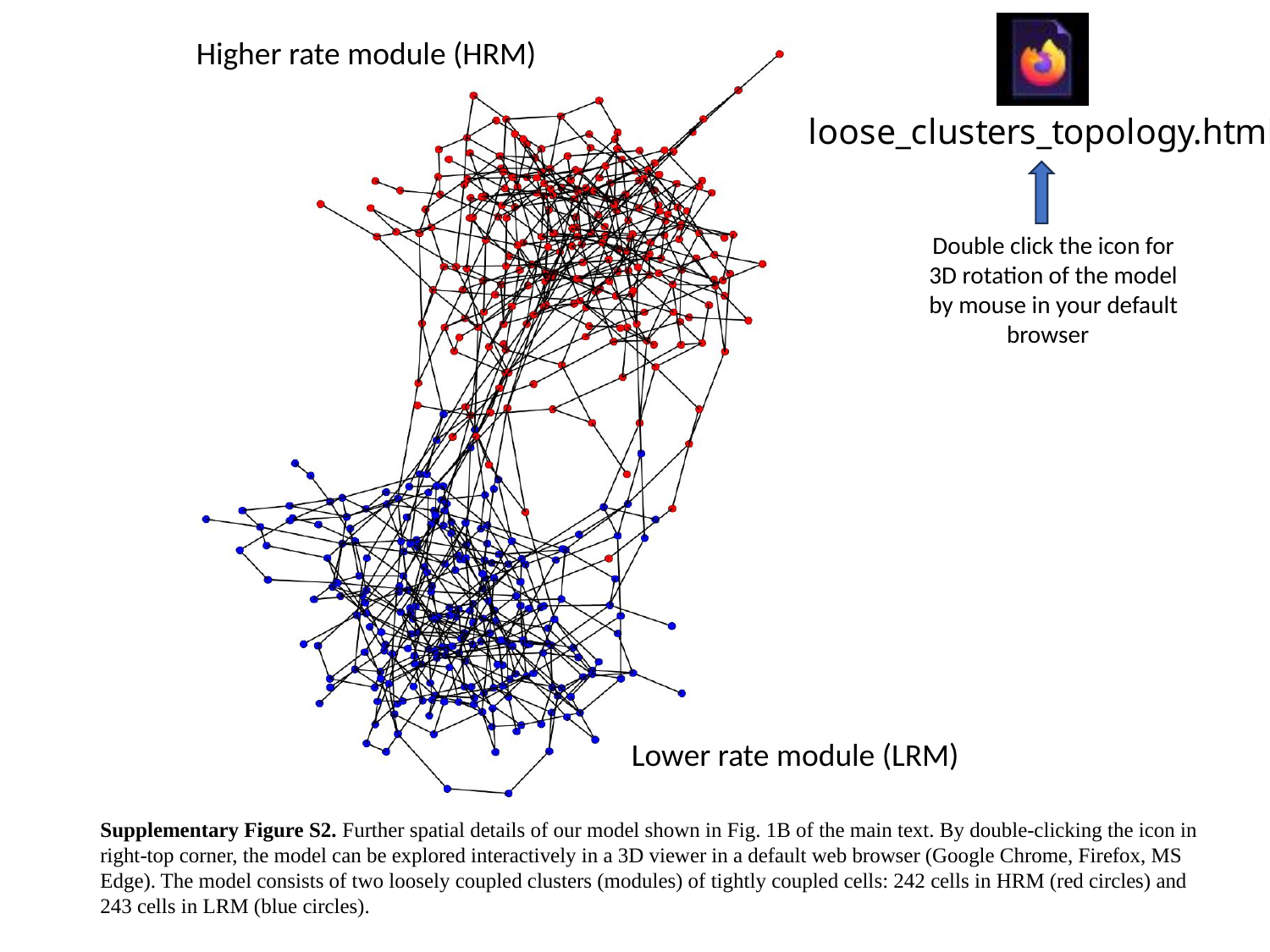

Higher rate module (HRM)
Double click the icon for 3D rotation of the model by mouse in your default browser
Lower rate module (LRM)
Supplementary Figure S2. Further spatial details of our model shown in Fig. 1B of the main text. By double-clicking the icon in right-top corner, the model can be explored interactively in a 3D viewer in a default web browser (Google Chrome, Firefox, MS Edge). The model consists of two loosely coupled clusters (modules) of tightly coupled cells: 242 cells in HRM (red circles) and 243 cells in LRM (blue circles).

## Slide 3
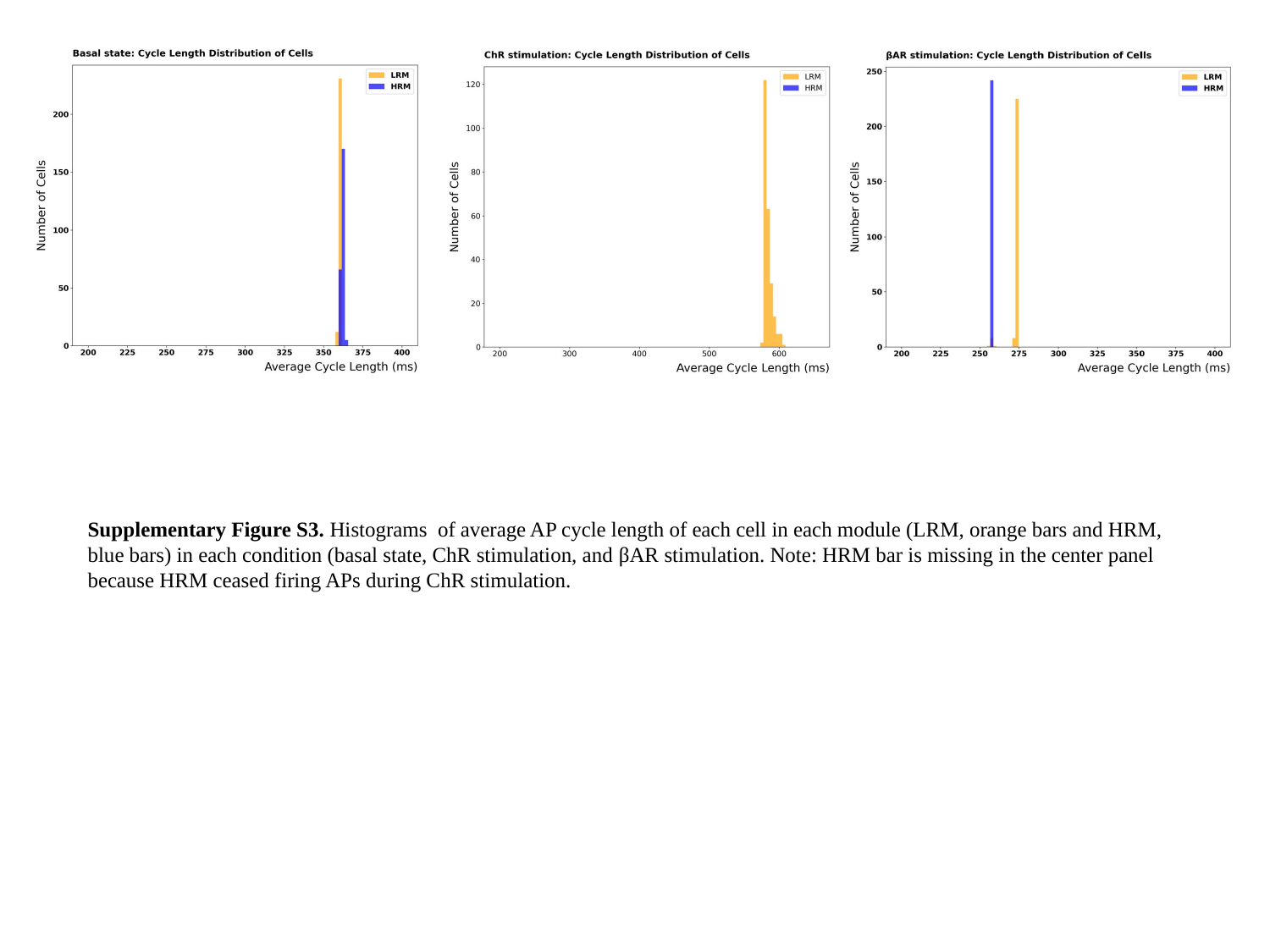

Supplementary Figure S3. Histograms of average AP cycle length of each cell in each module (LRM, orange bars and HRM, blue bars) in each condition (basal state, ChR stimulation, and βAR stimulation. Note: HRM bar is missing in the center panel because HRM ceased firing APs during ChR stimulation.

## Slide 4
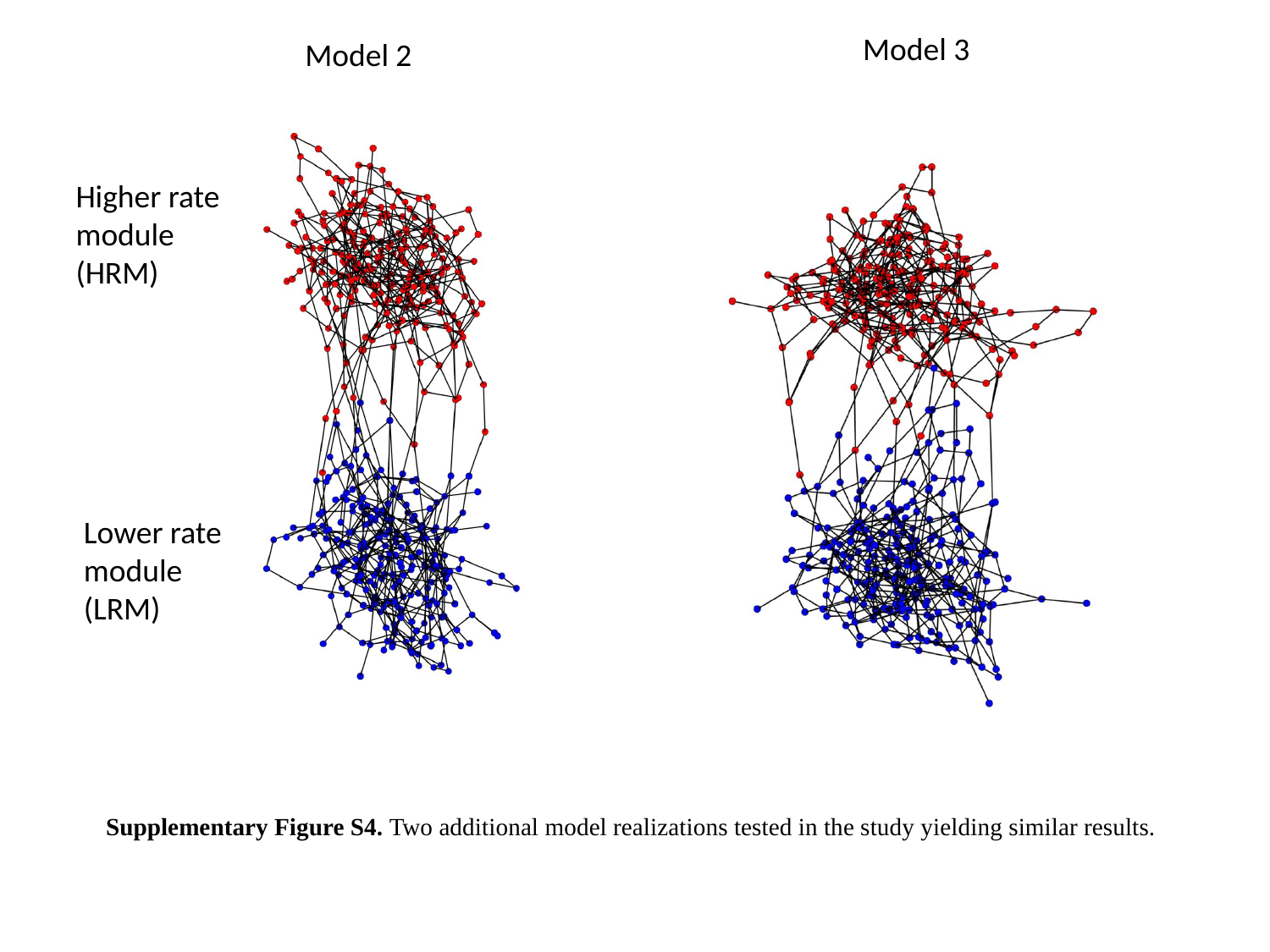

Model 3
Model 2
Higher rate module (HRM)
Lower rate module (LRM)
Supplementary Figure S4. Two additional model realizations tested in the study yielding similar results.

## Slide 5
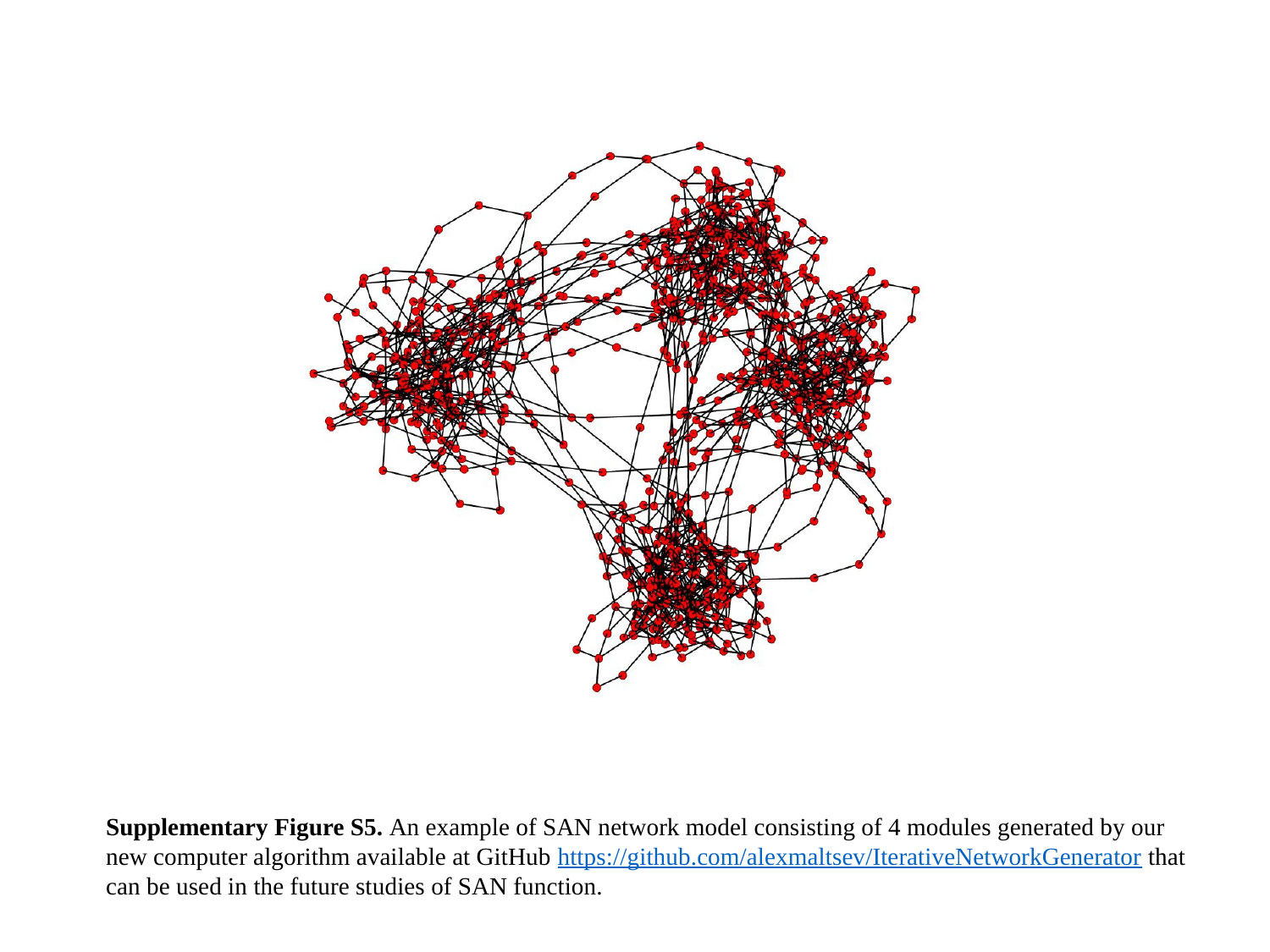

Supplementary Figure S5. An example of SAN network model consisting of 4 modules generated by our new computer algorithm available at GitHub https://github.com/alexmaltsev/IterativeNetworkGenerator that can be used in the future studies of SAN function.
